# The evolution of hierarchical structure building capacity for language and music: a bottom-up perspective

**DOI:** 10.1007/s10329-021-00905-x

**Published:** 2021-04-11

**Authors:** Rie Asano

**Affiliations:** Systematic Musicology, Institute of Musicology, University of Cologne, Cologne, Germany

**Keywords:** Comparative research, Evolution, Language, Music, Hierarchical structure building, Working memory

## Abstract

A central property of human language is its hierarchical structure. Humans can flexibly combine elements to build a hierarchical structure expressing rich semantics. A hierarchical structure is also considered as playing a key role in many other human cognitive domains. In music, auditory-motor events are combined into hierarchical pitch and/or rhythm structure expressing affect. How did such a hierarchical structure building capacity evolve? This paper investigates this question from a bottom-up perspective based on a set of action-related components as a shared basis underlying cognitive capacities of nonhuman primates and humans. Especially, I argue that the evolution of hierarchical structure building capacity for language and music is tractable for comparative evolutionary study once we focus on the gradual elaboration of shared brain architecture: the cortico-basal ganglia-thalamocortical circuits for hierarchical control of goal-directed action and the dorsal pathways for hierarchical internal models. I suggest that this gradual elaboration of the action-related brain architecture in the context of vocal control and tool-making went hand in hand with amplification of working memory, and made the brain ready for hierarchical structure building in language and music.

## Introduction

**Fig. 1:**
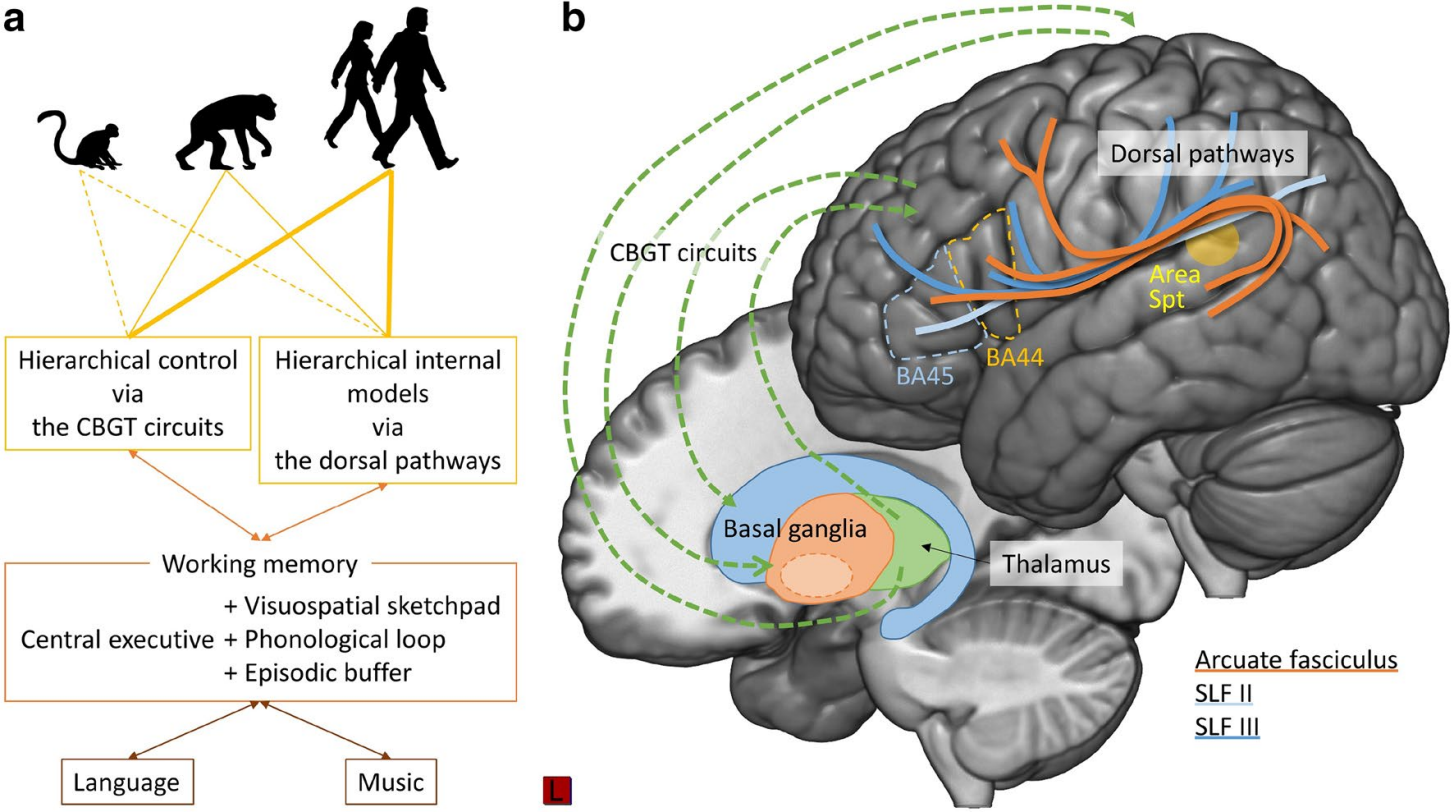
**a** Key idea of the current paper. Hierarchical control of goal-directed actions and hierarchical internal models are two central components of hierarchical structure building capacity. The basic brain architecture is shared between nonhuman primates and humans, but has gradually elaborated in the course of the evolution (yellow lines). This elaboration went hand in hand with the elaboration of working memory. Domain-specific representations of language and music emerged as a working memory resource-freeing strategy. **b** Brain structures central to the current paper. This figure displays the CBGT circuits (green dashed arrows), the dorsal pathways (arcuate fasciculus: orange line; SLF II: light blue line; SLF III: blue line), Broca’s region (BA44 and BA45), and the area Spt (yellow). The brain image was created using a Montreal Neurological Institute (MNI) template provided by MRIcrogl (https://www.mccauslandcenter.sc.edu/mricrogl/). The dorsal pathways were drawn based on Petrides (2014). Abbreviations: area Sylvian parietal-temporal (area Spt); cortico-basal ganglia-thalamocortical circuits (CBGT circuits); superior longitudinal fasciculus (SLF)

Language and music as cognitive systems form a mosaic consisting of multiple components as parts with different evolutionary origins (Fitch 2006; Boeckx 2013). From a comparative language-music perspective, some of these components might be shared and based on the same evolutionary genesis, while others might be different and emerged independently in the course of evolution. To date, several candidates for shared and distinct components have been proposed in both theoretical and empirical research (e.g., Patel 2008, 2012, 2013; Jackendoff 2009; Koelsch 2012; Peretz 2013; Asano and Boeckx 2015). One candidate shared component with the same evolutionary genesis is the capacity of hierarchical structure building. While some researchers deny the possibility of investigating the evolution of biological capacity underlying human cognitive systems by studying nonhuman animals (Hauser et al. 2014), the others emphasize a set of cognitive capacities that shed light on the evolution of uniquely human and domain-specific capacities (Boeckx 2017; Fitch 2017). This paper adopts the latter bottom-up perspective and surveys how the continuum between nonhuman primates’ cognitive capacities and human hierarchical structure building capacity looks like (Wakita 2020 for a similar approach). This work builds on my earlier proposal to investigate syntax in language and music in terms of the basic action-related components such as goal, planning, control, and sensory-motor integration (Asano and Boeckx 2015) and extends to a working memory framework for comparative evolutionary research. The key idea of the current paper as well as the central brain structures are summarized in Fig. 1.

## Hierarchical structure building from an action-oriented perspective

Language and music rely on three subcomponents of hierarchical structure building. The first subcomponent deals with a layered hierarchical structure with different levels of abstraction. Concerning language, phonemes combine into syllables, syllables combine into words, words combine into sentences, and sentences combine into discourses. As for music, pitch or sound events are hierarchically combined into larger units to encode affect, i.e., tension-relaxation patterns (Lerdahl and Jackendoff 1983). The second subcomponent deals with asymmetrical headed hierarchy. The linguistic syntactic structure consists of phrases with a head component determining the category of each phrase (e.g., a verb is the head of a verb phrase) and a non-head element called “complement.” In musical structure, the head is the structurally most important event of a musical unit determined by rhythmic and tonal-harmonic stability, and the non-head element called “elaboration” is a less important event (Lerdahl and Jackendoff 1983). There is a considerable difference between language and music in the way how the head is determined (Jackendoff 2009; Asano and Boeckx 2015), but the fact that they are both organized asymmetrically is a crucial similarity. The third subcomponent deals with hierarchically structured long-term memory representations called “rules,” “constructions,” “templates,” or “schemas.” The long-term memory representations are domain-specific and thus can be understood as the primary source of differences between language and music (Patel 2008). They are associated with meaning in language and with affect in music.

Hierarchical structures of language and music parallel action syntax which yields flexible action organization by building asymmetrical headed hierarchical structures and layered hierarchical structures. The former aspect of action syntax deals with the hierarchical combination of preparation and goal (i.e., head) for action planning (Jackendoff 2009). The latter deals with the hierarchical combination of lower-level goals (e.g., filling a pot with water, placing a filter in a machine) into higher-level goals (e.g., filling a machine with water, filling the filter with grained coffee) to achieve the main goal (e.g., making coffee). The higher the hierarchical level is, the more abstract and temporally extended the goals become. The concept of action syntax and its relation to syntax in language and music can be traced back to the seminal work of Lashley (1951) arguing against associative chain theories and proposing a hierarchical model of action sequencing as an alternative. He also suggested that hierarchical organization is characteristic of all skilled acts, including language and music.

Despite this striking parallel between the hierarchical structure in language, music, and action, research on the evolution of language and music, which takes the action-related components into account, rather focused on speech and beat-based timing (with an important exception of Fitch and Martins 2014). In particular, the action simulation for auditory perception (ASAP) hypothesis (Patel and Iversen 2014) and the gradual audiomotor evolution (GAE) hypothesis (Merchant and Honing 2014) have advanced this research area from an evolutionary neuroscience perspective. The ASAP hypothesis emphasizes the dorsal auditory pathway (SLF II) via the parietal cortex for beat processing and suggests that this pathway (especially its temporoparietal division of the SLF II) was enhanced in humans due to vocal learning (see also Cannon and Patel 2021 for a recent elaboration). In contrast, the GAE hypothesis claims that beat-based timing gradually evolved in primate lineage independently of vocal learning and emphasizes the role of the motor cortico-basal ganglia-thalamocortical (CBGT) circuit as a neural basis of timing shared between nonhuman primates and humans. It hypothesizes that in the course of the evolution in the primate lineage, the auditory system has gained increasingly privileged access to the motor CBGT circuit via the direct projection from the primary auditory cortex to the basal ganglia or via the dorsal auditory pathways.

The central claim which I would like to put forward here is that hierarchical structure building capacity for language and music builds on the same action-related neural architecture suggested by the ASAP hypothesis and the GAE hypothesis. The CBGT circuits and the dorsal pathways provide a foundation of hierarchical structure building: hierarchical control of goal-directed action (Badre and Nee 2018) and hierarchical internal models (Wolpert et al. 2003), respectively. In either case, hierarchy deals with different degrees of abstraction. The hierarchical organization of action implies that understanding and executing action sequences require hierarchical control, i.e., maintenance, selection, and inhibition of goals at differently abstract levels as well as the top-down control from more abstract onto more concrete levels (Badre 2008). The low-level goals directly correspond with concrete acts, while the higher-level goals are temporally extended and only indirectly govern concrete acts. The hierarchical organization of action also implies that, at multiple levels of abstraction, forward models should predict sensory consequences of actions, and inverse models should determine motor command based on desired outcomes (Wolpert et al. 2003). The lower-level internal models deal with concrete sensory consequences and motor commands, while the higher-level internal models deal with more abstract features. The interaction between hierarchical control and hierarchical internal models enables hierarchical structure building in sequencing.

Hierarchical control and hierarchical internal models are important constituents of hierarchical structure building capacity for language and music. For example, Hickok (2012) proposed the hierarchical state feedback control (HSFC) model, which investigates speech motor control as a hierarchical organization of internal models. The HSFC model includes phoneme-level, syllable-level, word-level, and conceptual-level internal models, which interact with each other. Bornkessel-Schlesewsky and colleagues (2015) suggested that a hierarchical organization of internal models can be also applied for processing linguistic sequences with various hierarchical levels including sentence-level and discourse-level. Prediction signals of the higher-level forward models propagate to the lower levels from top-down, while the lower-level error signals propagate to the higher levels to train the inverse models from bottom-up. Concerning music, Koelsch and colleagues (2019) proposed a similar hierarchical generative model of prediction based on predictive coding/active inference. Proksch and colleagues (2020) also interpreted beat-based timing from a perspective of predictive coding/active inference. As for hierarchical control, both language and music engage frontal goal hierarchies and the CBGT circuits (Ullman 2006; Kotz et al. 2009; Jeon 2014; Jeon and Friederici 2015; Lieberman 2016; Asano 2019). Thus, hierarchical control and hierarchical internal models provide a basis for hierarchical structure building in language and music.

## Building blocks for hierarchical structure building capacity in nonhuman primates

The bottom-up approach based on the action-related components makes the evolution of hierarchical structure building capacity for language and music tractable in terms of the continuity between nonhuman primates and humans. As I will argue below, nonhuman primates’ auditory-motor and visuomotor systems are equipped with the basic architecture for the interplay between hierarchical control and internal models, but are not fully expanded to account for more complex hierarchical structure building seen in language and music. The limitation is more evident in their auditory-motor systems than in visuomotor systems. In the latter case, some degree of hierarchical processing exists. Moreover, I argue that research on hominin tool-making contributes to the research on how the action-related neural architecture was gradually elaborated in the hominin evolution.

### Orofacial and vocal control

Nonhuman primates possess a set of prerequisites for a component necessary for hierarchical structure building in speech (or phonology), namely the capacity for synchronized and voluntary orofacial and vocal control generating a sequential vocal stream. Flexible, modifiable, and variable orofacial and vocal behavior is present in some nonhuman primates although they do not belong to the “classical” vocal learners (for discussions on vocal learning in primates, see Lameira 2017; Fischer and Hammerschmidt 2020; Martins and Boeckx 2020).

For example, following MacNeilage’s (1998) frame/content theory, Ghazanfar and Takahashi (2014a, b) point to monkey lip-smacking (facial expression including oscillatory jaw movement) and call (vocal expression) as precursors for speech and propose the synchronization between facial and vocal expression as the babbling-like first step for the emergence of speech featuring syllable-like sequences in the hominin line. They point out that the evolution of the synchronization between facial and vocal expressions as audiovisual communication signals may be simple because the gelada baboon, a unique nonhuman primate species showing such a synchronization known as a wobble, is closely related to the yellow baboon not displaying anything resembling a wobble. Recently, Risueno-Segovia and Hage (2020) showed that marmoset monkeys display synchronized jaw movement and vocalization in producing phee calls, providing evidence for early emergence of synchronized orofacial and vocal control yielding a sequential vocal stream. However, its neural mechanism remains unrevealed. In humans, Brown and colleagues (2020) determined that somatotopic representations of the larynx and jaw muscles overlap in the primary motor cortex, which could be one of the candidates making such a synchronization possible.

In addition to synchronized orofacial and vocal control, researchers identified voluntary vocal control as an ability required for generating speech-like sequential vocal streams. The ventrolateral prefrontal cortex of nonhuman primates also plays a crucial role in voluntary vocal control. In their review, Hage and Nieder (2016) provided evidence that the monkey’s ventrolateral prefrontal cortex is associated with the preparation for vocalization. In particular, macaque area 44, which is comparable to human BA44, was found to be involved with the orofacial musculature (Petrides et al. 2005) and is highly connected with the larynx motor cortex (Kumar et al. 2016). Loh and colleagues (2017) reviewed the distinctive role of the ventrolateral frontal cortex, the anterior/mid-cingulate cortex, and the (pre-)supplementary motor area of humans and nonhuman primates in the voluntary vocal control: the ventrolateral frontal cortex is associated with the selection of orofacial-vocal responses based on sensory-motor conditional rules (i.e., IF Stimulus A, THEN Vocalization A); the mid-cingulate cortex is involved in evaluating consequences of the responses; and the pre-supplementary motor area is associated with the general initiation and adjustment of the responses. Thus, although preparation for vocalization is more effortful than for action execution in macaques (Koda et al. 2018), the basic mechanism underlying voluntary vocal control seems to be present.

Although some homologous mechanism is present in nonhuman primates, the human voluntary vocal control mechanism contains at least three major novelties in comparison to that of nonhuman primates. The first is the direct cortico-ambigual connection which is absent in monkeys, sparse, if at all, in chimpanzees, and enhanced in humans (Kuypers 1958a, b; Jürgens 2002). It was hypothesized to have emerged through an exaptation of the corticospinal tract subserving manual motor control (Fitch 2011). The second is the two-part structure of the human larynx motor cortex consisting of the ventral larynx cortex, which is homologous to that of monkeys and chimpanzees, and the uniquely human dorsal larynx cortex which was hypothesized to have emerged through the duplication of either vocalization-related motor areas or adjacent non-vocal motor areas (Belyk and Brown 2017). It is also the dorsal larynx motor cortex that overlaps with the jaw motor cortex (Brown et al. 2020). The third is increased connectivity of the human dorsal larynx cortex with structures in the parietal lobe including the somatosensory cortex and the inferior parietal lobe, while the structural networks of the larynx motor cortex in monkeys and humans are mainly similar otherwise (Kumar et al. 2016). Those changes may have elaborated basic mechanisms shared among humans and nonhuman primates to realize hierarchical planning and control of vocalization.

Recently, by referring to the study conducted by Kumar and colleagues (2016), Hickok (2017) argued that the dorsal stream including the area Spt (Sylvian parietal-temporal) elaborated gradually as a sensory feedback control circuit in the context of the evolution of voluntary laryngeal control. The area Spt is a crucial part of the dorsal auditory pathway and was suggested to be an auditory-motor interface system (Hickok and Poeppel 2004, 2007). Aboitiz (2017, 2018) sees the evolution of the dorsal pathways linking to the posterior Broca’s region as a continuum of this elaboration. Dorsal auditory pathways—although sparse—are already present in macaques (Petrides 2014; Balezeau et al. 2020) and show a gradual expansion via chimpanzees to humans (Rilling et al. 2008; Balezeau et al. 2020). In humans, the dorsal pathways were suggested to implement the sensory-motor prediction and feedback control at differently abstract hierarchical levels (Bornkessel-Schlesewsky et al. 2015; Hickok 2017). While the auditory dorsal pathways of nonhuman primates and humans implement internal models, hierarchical levels of internal models are limited in nonhuman primates (Bornkessel-Schlesewsky et al. 2015). Such a hierarchical organization of internal models may go hand in hand with hierarchical chunking implemented in the CBGT circuits (Rauschecker 2012). Macaques show activations in the frontal lobe, the parietal and temporal lobe, and the basal ganglia in processing auditory chunks, indicating auditory-motor integration (Uhrig et al. 2014). However, the hierarchical levels of chunks seem to be more limited in nonhuman primates than in humans (Merchant and Honing 2014). In this way, the gradual elaboration of the dorsal auditory pathway together with the CBGT circuits could have led to the hierarchical vocal control.

### Action, tool use, and tool-making

Research on planning and control provides an optimal opportunity to study the continuity of hierarchical structure building capacity in humans and nonhuman primates, as the rich capacity for action planning and control is well known in nonhuman primates. For example, Weiss and colleagues (2012) argued that cotton-top tamarin monkeys and lemurs are capable of short-span anticipatory planning as they adjust their hand posture for grabbing a cup in advance according to the affordance of the cup. Wynn and colleagues (2011) pointed out that, in addition to great apes, the long-tailed macaque and the bearded capuchin use tools to extract food that is otherwise not accessible. Although they do not discuss tool use in terms of planning, tool use as such can be regarded as involving at least one preparatory action: grasping a stone (i.e., preparation) to crack a nut (i.e., head). Byrne and colleagues (2013) reviewed evidence for chimpanzee’s planning capacity concerning tool use in the wild: chimpanzees manufacture and repair tools or pick up suitable materials in advance. This indicates that chimpanzees can prepare for the main action in a more extended time range.

Action planning and control of nonhuman primates are hierarchically organized. Byrne and colleagues (2013) argued that chimpanzee’s tool use is hierarchically organized because they can flexibly omit redundant steps in the tool-use sequence to achieve the goal of fishing termites. Byrne and Russon (1998) showed the hierarchical organization of a mountain gorilla’s food preparation and an orangutan’s imitation. They suggested that great apes can represent and manipulate the relationship between objects at different hierarchical levels, although the hierarchical depth of planning is limited. Matsuzawa (1996) showed the hierarchical organization of chimpanzees’ tool-use behavior. He suggested nut-cracking behavior which includes the use of a wedge stone as the most complex form of tool use in wild chimpanzees with nested hierarchical levels. The wedge stone supports an anvil stone on which the nut is placed and hit by a hammerstone. Matsuzawa (1991) and Hayashi (2007) demonstrated that captive chimpanzees can hierarchically combine multiple cups. All those examples can be interpreted such that great apes are able to organize action into hierarchical structures consisting of preparation and the head.

Archaeological evidence suggests a gradual elaboration of action syntax in the hominin evolution. For example, Moore (2010) showed that early stone tool-making solely required serial flaking to remove high mass from the core, while hierarchical flaking with (multiple) preparatory steps is necessary for Acheulean and Lavallois tool-making. Although Stout (2011) also ascribes hierarchical organization to early stone tool-making in the Oldowan industry, it can be claimed that hierarchical organization is not necessary for Oldowan tool-making given the absence of preparatory flaking. His research shows that hierarchical flaking with a preparatory step emerged in early Acheulean tool-making and was elaborated through additional hierarchical levels in late Acheulean and Lower Palaeolithic tool-making. Stout and colleagues (2018) also showed that Acheulean tool-making is computationally more demanding than Oldowan tool-making. Ambrose (2001) even suggested that neither Oldowan nor Acheulean tool-making, but Middle Paleolithic/Middle Stone Age tool-making by Neanderthals, late archaic humans, and anatomically modern humans yielding composite tools is hierarchical. He suggested that combining technological units such as a shaft, a stone insert, and binding materials in different configurations generates functionally different tools.

Evidence from paleoneurology investigating hominin endocranial morphology suggests a series of neuroanatomical changes. Holloway (2015) suggests the relative expansion of the posterior parietal association cortex at the cost of the visual cortex approximately from 3.5 to 3.0 million years ago (Australopithecus) and the reorganization of the Broca’s region approximately 1.8 million years ago (*Homo rudolfensis*). Bruner (2004, 2010) further suggests the widening of the frontal cortex as one change that happened in Neanderthals and Homo sapiens, but globularity and the general enlargement of the entire parietal surface as traits unique to Homo sapiens and absent in adult Neanderthals. Globularity is associated with parietal and cerebellar bulging (Neubauer et al. 2018).

Although they are general neuroanatomical changes and cannot be directly linked to the gradual elaboration of action syntax, there is at least evidence that some of those structures change in the context of tool use and tool-making. The parietal cortex shows tool-use-induced expansion in monkeys (Quallo et al. 2009). There is a heritable link between the individual variation in tool-use capacity and the morphology in temporal, parietal, and cerebellar cortices of chimpanzees (Hopkins et al. 2019). Cerebellum size correlates more strongly with foraging skills than social group size, while neocortex size shows the reverse correlation pattern (Barton 2012). Moreover, Hecht and colleagues (2015b) showed that Paleolithic tool-making training remodels frontoparietal circuits in humans. Neuroimaging studies investigating stone tool-making in humans showed that the posterior parietal region plays a significant role in sensory-motor integration required for Oldowan flaking (Stout and Chaminade 2007; Stout et al. 2008). Additional right inferior frontal gyrus activation is significant for hierarchical control required for Acheulean flaking (Stout et al. 2008, 2011; Putt et al. 2017).

In humans, hierarchical control recruits the frontal lobe, the parietal lobe, the basal ganglia, and the cerebellum (Balleine et al. 2015; Badre and Nee 2018; Badre and Desrochers 2019; D’Mello et al. 2020). Both in humans and nonhuman primates, the frontal lobe is organized to form the rostrocaudal abstraction gradient: the more caudal region processes more concrete sensory-motor representation, while the more rostral region more abstract representation (Badre and D’Esposito 2009). The frontal lobe of humans and nonhuman primates projects massively to the basal ganglia which play a crucial role in goal-directed (i.e., reward-based) control and learning (Alexander et al. 1986; Middleton and Strick 2000b; Haber 2003, 2016). The cerebellum of humans and nonhuman primates works in tandem with the frontal lobe, the basal ganglia, and the parietal lobe, and plays a crucial role in building internal model and optimizing behavior (Wolpert et al. 1998; Middleton and Strick 2000a; Ramnani 2006; Ito 2008; Bostan and Strick 2018). Thus, the basic brain architecture underlying action syntax is the same for humans and nonhuman primates.

One prominent difference in the hierarchical control network is one of the frontoparietal circuits, namely the superior longitudinal fasciculus (SLF III), which is right-lateralized and larger, and projects more strongly to the inferior frontal gyrus in humans than in chimpanzees (Hecht et al. 2015a). Fronto-parieto-temporal connections via the superior and middle longitudinal fasciculi are more dominant in humans, while frontotemporal connections via the extreme/external capsules are more prominent in macaques, and the fronto-parieto-temporal connectivity profile in chimpanzees is intermediate (Hecht et al. 2013). The SLF III as a dorsal pathway enables integration of complex goals represented in the frontal lobe and increasingly complex sensory-motor representations in the parietal lobe (Stout and Hecht 2017), which supports increasingly complex hierarchical structure building through hierarchical internal models (see Wolpert et al. 2003 for hierarchical internal models). In this way, the gradual elaboration of the SLF III could have led to the amplification of action syntax.

## Integration into a working memory framework for comparative evolutionary study

In Sects. 2 and 3, I argued that the evolution of hierarchical structure building capacity for language and music can be studied based on the action-related components, and the basic brain architecture for the action-related components is shared between humans and nonhuman primates. In the current section, I suggest that the gradual elaboration of the action-related brain architecture in the context of vocal control and tool-making went hand in hand with amplification of working memory, and made the brain ready for hierarchical structure building in language and music.

Working memory is not a passive store, but an active information processing device consisting of multiple components including central executive, visuospatial sketchpad, phonological loop, and episodic buffer (for reviews, see Baddeley 2010, 2012). The central executive is an attentional control system that is supported by two short-term maintenance systems: the visuospatial sketchpad for visual information and the phonological loop for verbal and acoustic information. Each of them contains two components: a store (also buffer) that records (sensory) memory traces and a (covert motor) rehearsal process that refreshes and maintains those traces. That is, both visuospatial sketchpad and phonological loop can be conceived as interfaces for sensory-motor integration. The episodic buffer is a short-term store holding multimodal memory units, provides a platform where different components of working memory interact, and interfaces with long-term memory. Importantly, working memory is a fluid system requiring only temporary activation, while long-term memory is a crystallized system representing skills and knowledge.

Gradually increasing complexity in tool-making went hand in hand with the elaboration of the visuospatial sketchpad and the central executive. For example, Oldowan flaking requires sophisticated visuomotor coordination through more than a few hours of training (Stout 2010; Stout and Chaminade 2012), and its accuracy differs between experts and novices (Bril et al. 2010; Nonaka et al. 2010). That is, the integration of visuospatial and motor representations through the visuospatial sketchpad is central to Oldowan tool-making. The maintenance of stable visual representations through the visuospatial sketchpad is crucial for tool-making in general in learning to associate motor control parameters and visual consequences (Coolidge and Wynn 2005). Moreover, the emergence of explicit preparatory steps, which can be embedded in another preparatory action, in Acheulean tool-making by *Homo erectus* (Moore 2010; Stout 2011) implies a need for more sophisticated planning ability as well as the ability to maintain and manipulate temporarily extended goal and subgoal representations in the mind. Finally, Middle Paleolithic/Middle Stone Age composite tool-making requires long-range planning and coordination of multiple task sets (Ambrose 2001). That is, the central executive gradually elaborated to control, i.e., select and inhibit, representations maintained at multiple hierarchical levels and in multiple task sets.

Further, I propose that the homologous neural circuits for auditory-vocal-orofacial control present in nonhuman primates provide a basis for the phonological loop. Although there is evidence that auditory long-term and short-term memory are limited in monkeys (e.g., Fritz et al. 2005; Scott et al. 2012), a recent review points to the similarity of auditory memory storage in humans and nonhuman primates (Scott and Mishkin 2016). Moreover, nonhuman primates can process auditory sequences with nonadjacent dependency (e.g., AB^n^A) which requires a long-term memory representation of a canonical auditory pattern and an auditory storage capacity to hold an element over one or more intervening elements and compare it to another element (for reviews, Wilson et al. 2017, 2020; Petkov and ten Cate 2020). Thus, the emphasis was put on the rehearsal mechanism mapping sounds to articulatory movement via the elaborated dorsal auditory pathways rather than on the auditory memory (Aboitiz et al. 2006, 2010; Scott and Mishkin 2016; Aboitiz 2017, 2018). The phonological loop as an auditory-motor loop is based on an internal model: a forward model from the frontal cortex to the temporal cortex predicts the auditory consequence of an action, an inverse model from the temporal cortex to the frontal cortex transforms desired auditory outcome into motor command, and the area Spt serves as an auditory-motor interface (Buchsbaum and D’Esposito 2019).

Aboitiz and his colleagues argued that an enhanced capacity of the phonological loop in humans was a key to learn and process complex hierarchical sequences with multiple embeddings (Aboitiz et al. 2006, 2010). Coolidge and Wynn (2007) made a similar suggestion. However, the increased maintenance capacity alone does not lead to hierarchical structure nor embedding. Alternatively, I suggest that hierarchical structure relates rather to a memory-optimization strategy. As a short-term memory component, the phonological loop has a limited maintenance capacity of approximately three to five elements (Cowan 2010). Thus, this limited capacity should be optimally used in processing sequences. Chunking, i.e., grouping multiple elements as a unit, is a strategy to optimize memory resource use and can be regarded as compression of information at multiple hierarchical levels (Snyder 2016; Christiansen and Chater 2016). The higher the levels of chunks are, the more abstract the representations become. Rules, then, represent the (hierarchical) relationship between chunks and allow for more complexity in sequencing once stored in the long-term memory. Petkov and Wilson (2012) proposed that selective pressures to reduce memory demands through rule-based learning strategies may have expanded human hierarchical structure building capacity. Coolidge and Wynn (2005) point out the importance of learned rules stored in long-term memory for freeing up working memory capacity to access and manipulate specific content.

In comparative language-music research, working memory was suggested as a candidate shared mechanism underlying hierarchical structure building in language and music, as it is required for maintaining and manipulating intermediate results (Kljajevic 2010; Fitch and Martins 2014). Building up multiple parallel hierarchical structures, hierarchical structures with multiple embedding, or hierarchical structures deviating from canonical long-term memory representations leads to interference effects between language and music processing as extraordinary demand is placed on the working memory resources. Once units and rules are stored in the long-term memory and hierarchical processing can be partially automatized, the processing load decreases. Thus, freeing of working memory resources is one possible reason for functional specialization within the shared brain architecture for different cognitive systems.

How does functional specialization emerge? This question, then, can be tackled within a domain-*relevant* approach. Its main idea is that through neural competition, brain networks become relatively domain-specific overtime (Karmiloff-Smith 2013). That is, specialization of function can be regarded as fine-tuning of coarsely coded systems with domain-relevant biases. For example, the CBGT circuits implement hierarchical structure building in language and music through specialized parallel subcircuits (Ullman 2006; Asano 2019). The dorsal auditory pathways also show specialization such that the left arcuate fasciculus plays a more important role for hierarchical structure building in language than in music, while the reverse is true for the right arcuate fasciculus (Friederici 2019). In this way, language and music can be regarded as different cognitive capacities that emerge from different uses of the same brain architecture. This is in line with the past theses introduced under the terms of modularization (Karmiloff-Smith 1992), neuronal recycling (Dehaene and Cohen 2007; Peretz et al. 2015), neural reuse (Anderson 2010), or neural retuning (Matchin 2018).

## Conclusions and future directions

This paper investigated the continuum between nonhuman primates’ cognitive capacities and human hierarchical structure building capacity, and integrated the findings of comparative research into a working memory framework. Hierarchical structure building requires working memory to maintain and manipulate intermediate results based on stored long-term memory representations. In particular, I argued that the evolution of hierarchical structure building capacity for language and music can be investigated in terms of the action-related components such as goal, planning, control, and sensory-motor integration by focusing on the hierarchical control of goal-directed action implemented in the cortico-basal ganglia-thalamocortical (CBGT) circuits and hierarchical internal models realized by the dorsal pathways. The basic brain architecture crucial for hierarchical structure building in language and music is shared with nonhuman primates, but has elaborated in the course of hominin evolution to accomplish more complex hierarchical structure building in sequencing. This elaboration went hand in hand with the enhancement of working memory. Moreover, I proposed that one possible explanation for functional specialization of the brain architecture for language and music is a working memory resource optimization strategy. This line of comparative research from a bottom-up perspective makes it possible to study the evolution of hierarchical structure building capacity for language and music. However, this paper still leaves several questions open for future research.

For example, as reviewed above, hominin tool-making underwent gradual elaboration of action syntax and thus neuroanatomical change could directly relate to the hierarchical complexity of tool-making. On the other hand, to learn such a complex procedure, more complex social learning ability including imitation and teaching is required (Morgan et al. 2015; Laland 2017; Stout and Hecht 2017). It is also possible that the difference in the SLF III could reflect the difference in the social learning capacity (Hecht et al. 2013, 2015a). To learn how to use and make a tool by imitation, for example, one’s own internal model should be linked to the state of the other, i.e., a simulation of the observed percept through one’s own internal model is required (Wolpert et al. 2003; Stout and Hecht 2017). One possibility to disentangle this issue is comparative neuroanatomy of chimpanzee and bonobo (see, for example, Rilling et al. 2012): the former is a good tool user with less degree of prosociality, while the latter displays a high degree of prosociality and can socially learn tool-making, but rarely shows tool use in the wild (Wynn et al. 2011; and Gruber and Clay 2016 give good summaries of bonobo’s tool-making ability). Therefore, research on action syntax also makes it possible to study why the basic brain architecture (or one particular subcomponent of it) crucial for hierarchical structure building was elaborated to yield more complex behavior.

Moreover, the bottom-up approach of this paper can also profit much from the progress made in the research on vocal learning across species (e.g., Petkov and Jarvis 2012; Lattenkamp and Vernes 2018; Jarvis 2019; Martins and Boeckx 2020). In particular, research on songbirds could contribute to revealing the brain mechanisms underlying chunking and rule-learning. In learning songs, juvenile songbirds break tutor songs into smaller units, produce those units as chunks to practice singing, and recombine those chunks to create their songs with large individual repertoires (Marler 2000; ten Cate and Okanoya 2012). Thus, chunks are units of manipulation in song production and perception as well as rule-learning. In non-vocal learning species, gibbons could be an additional model to investigate chunk-based vocal control as they show chunk-based organization in their song-like vocalization (Inoue et al. 2017).

Finally, the brain architecture discussed in the current paper points to a particular computational neurocognitive modeling strategy, namely hierarchical reinforcement learning, which unifies hierarchical control of goal-directed action and hierarchical internal models (e.g., Haruno et al. 2003; Frank and Badre 2012; Alexander and Brown 2018). This modeling strategy in combination with the evolutionary simulation of recursive combination as suggested by Toya and Hashimoto (2018) could provide us with a possibility to investigate why hierarchical structure building capacity has evolved in its full range of complexity in the hominin line. This allows language and music evolution research to move toward an integrated approach discussed elsewhere (Asano and Seifert 2018). Altogether, I hope the current paper made the evolution of hierarchical structure building capacity for language and music more tractable for comparative evolutionary research and offered possible future directions for interdisciplinary research.
